# Zoonotic Fecal Pathogens and Antimicrobial Resistance in Canadian Petting Zoos

**DOI:** 10.3390/microorganisms6030070

**Published:** 2018-07-16

**Authors:** Cheyenne C. Conrad, Kim Stanford, Claudia Narvaez-Bravo, Norman F. Neumann, Krysty Munns, Lisa Tymensen, Cassandra Jokinen, Tim A. McAllister

**Affiliations:** 1 Alberta Agriculture and Forestry, Lethbridge, AB T1J 4V6, Canada; cheyenne.conrad@agr.gc.ca (C.C.C.); kim.stanford@gov.ab.ca (K.S.); lisa.tymensen@gov.ab.ca (L.T.); cassandra.jokinen@gov.ab.ca (C.J.); 2 Canadian Association of Fairs and Exhibitions, Brandon, MB R7B 3W8, Canada; 3 Department of Food Science, University of Manitoba, Winnipeg, MB R3T 2N2, Canada; Claudia.NarvaezBravo@umanitoba.ca; 4 Department of Public Health, University of Alberta, Edmonton, AB T6G 2G7, Canada; nfneuman@ualberta.ca; 5 Agriculture and Agri-Food Canada, Lethbridge Research and Development Centre, Lethbridge, AB T1J 4B1, Canada; krysty.munns@agr.gc.ca

**Keywords:** petting zoo, zoonoses, pathogens, Shiga toxin-producing *Escherichia coli* (STEC), methicillin-resistant *Staphylococcus aureus* (MRSA), antimicrobial resistance

## Abstract

This study aimed to better understand the potential public health risk associated with zoonotic pathogens in agricultural fairs and petting zoos in Canada. Prevalence of *Salmonella*, Shiga toxin-producing *Escherichia coli* (STEC) O157:H7, and top six non-O157 STEC serogroups in feces (*n* = 88), hide/feather (*n* = 36), and hand rail samples (*n* = 46) was assessed, as well as distributions of antimicrobial resistant (AMR) broad and extended-spectrum β-lactamase (ESBL)-producing *E. coli*. Prevalence of methicillin-resistant *Staphylococcus aureus* (MRSA) in pig nasal swabs (*n* = 4), and *Campylobacter*, *Cryptosporidium*, and *Giardia* in feces was also assessed. Neither *Salmonella* nor MRSA were detected. *Campylobacter* spp. were isolated from 32% of fecal samples. *Cryptosporidium* and *Giardia* were detected in 2% and 15% of fecal samples, respectively. Only one fecal sample was positive for STEC O157, whereas 22% were positive for non-O157 STEC. Multi-drug resistance (MDR) to antibiotics classified as critically and highly important in human medicine was proportionally greatest in *E. coli* from cattle feces. The β-lactamase-producing *E. coli* from pig, horse/donkey feces, and hand rail samples, as well as the STEC *E. coli* from handrail swabs were MDR. The diversity and prevalence of zoonotic pathogens and AMR bacteria detected within agricultural fairs and petting zoos emphasize the importance of hygienic practices and sanitization with respect to reducing associated zoonotic risks.

## 1. Introduction

Agricultural fairs and petting zoos provide the public with the opportunity to interact and learn about farm animals. However, farm animals also present a risk for transmission of zoonotic pathogens [1]. Illnesses and outbreaks involving Shiga toxin-producing *Escherichia coli* (STEC) O157:H7 and other non-O157 serogroups, *Campylobacter* spp., *Salmonella* spp., *Cryptosporidium* spp., and to a lesser degree, *Listeria monocytogenes* and *Yersinia enterocolitica* have been reported in fair and petting zoo visitors [2,3]. It has been estimated that 14% of all illnesses caused by these pathogens arises from direct animal contact [3].

Humans can become infected with zoonotic pathogens by hand-to-mouth ingestion of animal feces or through direct contact with animals or contaminated surfaces [2,4]. The severity of illness can vary, but often results in self-limiting episodes of gastroenteritis characterized by abdominal cramps, nausea, vomiting, fever, and watery or bloody diarrhea which occasionally becomes hemorrhagic [5,6]. Severe cases can cause kidney damage or even death [5]. Studies have shown that petting zoo visitors frequently engage in practices that promote the transmission of pathogens such as touching their face after animal contact, letting animals lick their hands, or eating and drinking within animal enclosures [7]. Furthermore, several studies have found that hand-washing compliance by visitors is often poor ranging from 0% to 77% [1,7,8].

An additional concern for both human and animal health is the transmission of antimicrobial resistant (AMR) bacteria [9]. The emergence of bacteria resistant to “critically important” (e.g., third and fourth generation cephalosporins, streptomycin, ampicillin) or ‘highly important’ (e.g., sulfamethoxazole, trimethoprim, chloramphenicol, tetracyclines) antibiotics is of particular concern, as there are limited therapeutic options to treat infections caused by these bacteria [9,10,11]. Although generic *E. coli* are typically not pathogenic, they may harbor antibiotic resistance and virulence genes that can be transferred to other bacteria, including pathogens.

To better understand potential public health risks associated with petting zoos, the presence of the most prominent zoonotic pathogens associated with animal feces (i.e., STEC O157:H7, the top 6 non-O157 serogroups [12] O26, O45, O103, O111, O121, and O145, *Campylobacter*, *Cryptosporidium*, *Giardia*, and *Salmonella*), broad- and extended-spectrum β-lactamase (ESBL) resistant *E. coli*, as well as methicillin-resistant *Staphylococcus aureus* (MRSA) as indicator species for antibiotic resistance, were assessed in samples collected from animals and their environment at fairs and petting zoos in Canada. Though guidelines exist for maintaining hygienic and safe animal exhibits to reduce the risk of the public ingesting pathogens, there are no official regulations or inspections to enforce these measures. An online survey was distributed to livestock fair exhibitors across Canada to gain a broader understanding of the hygienic practices and safety precautions employed at these events.

## 2. Materials and Methods

### 2.1. Survey Data Collection

An online questionnaire was distributed to managers of 782 fairs and animal exhibitions across Canada (Enigma Research Corporation, Toronto, ON Canada). The survey included a variety of questions regarding the characteristics and cleaning practices of livestock pavilions and petting zoos at fairs.

### 2.2. Sample Collection and Preparation

**Figure 1:**
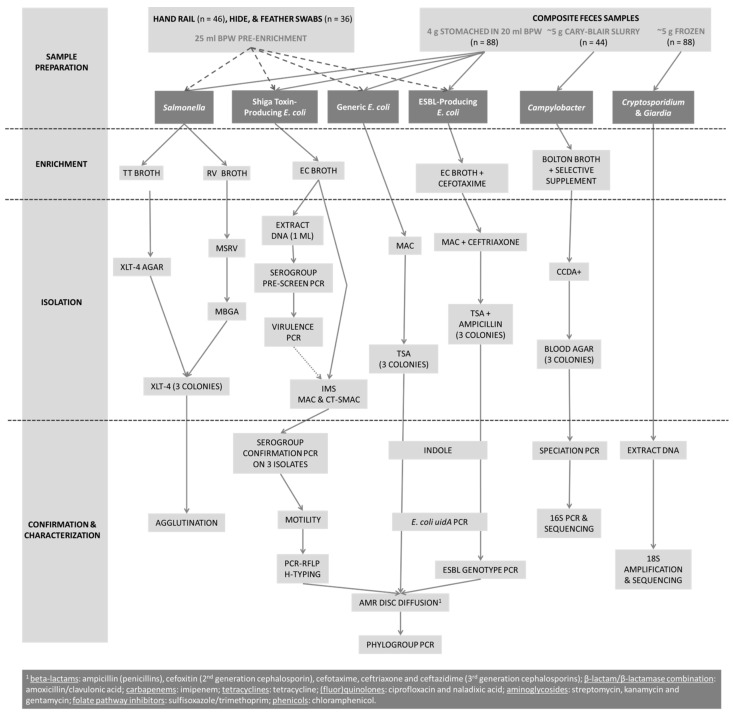
Flow chart representation of study methodology.

Over two consecutive years, hand rail swabs (*n* = 46), hide/feather swabs (*n* = 36), composite fecal samples (*n* = 88), and nasal swabs (*n* = 4) were collected from a permanent seasonal petting zoo and mobile petting zoos located in Alberta (*n* = 5) and Manitoba (*n* = 1). Samples were processed according to Figure 1. Hand rails (approximately 75 cm^2^) and animal hides/feathers (neck and back areas, 100 cm^2^) were swabbed with sterile, moistened sponges (3M, St. Paul, MN, USA) and placed in buffered peptone water (BPW; Hardy Diagnostics, Santa Maria, CA, USA). Fresh feces from cows, calves, donkeys, horses, sheep, goats, llamas, pigs, and birds (i.e., ducks, chickens, parakeets, and peacocks) were collected from pen floors, pooled (5 samples from different animals of the same species), and homogenized. A sub-sample was added to Cary-Blair medium (Dalynn Biologicals, Calgary, AB, Canada). Nasal swabs (BBL CultureSwab, Cary-Blair transport medium, BD Canada, Mississauga, ON, Canada) were only collected from pigs. Samples were maintained at 4 °C until processing.

In the laboratory, feces were frozen for *Cryptosporidium* and *Giardia* analysis. Sponge swabs in BPW were pre-enriched at 37 °C overnight (~18 h) for *Salmonella*, STEC, generic *E. coli*, and ESBL-producing *E. coli*. Feces from composite samples were homogenized in BPW for 1 min at 230 RPM using a Seward Model 400 stomacher (Cole-Parmer Canada, Montreal, QC, Canada).

### 2.3. Salmonella Screening

Alongside positive (*Salmonella* Typhimurium ATCC 14028) and negative (*E. coli* ATCC 25922) controls, feces and sponge swab washings were each dispensed into Tetrathionate (TT; BD Canada, Mississauga, ON, Canada) and Rappaport-Vassiliadis (RV; EMD Millipore, Etobicoke, ON, Canada) broths and incubated at 42 °C for 20–24 h. Tetrathionate enrichments were streaked onto Xylose Lysine Tergitol 4 agar (XLT-4; BD Canada, Mississauga, ON, Canada) and RV enrichments were transferred to modified semisolid RV (MSRV) agar plates and incubated at 37 °C for 24 h. A loop of MSRV culture from the edge of opaque zones was streaked onto modified Brilliant Green agar (MBGA; Hardy Diagnostics, Santa Maria, CA, USA) and incubated overnight at 37 °C for 24 h. Up to three colonies per XLT-4 and MBGA plate were sub-cultured to fresh XLT-4 plates and incubated at 37 °C for 24 h. Presumptive *Salmonella* isolates were tested for agglutination with Poly A-I + Vi antiserum (BD Canada, Mississauga, ON, Canada).

### 2.4. STEC Screening

Feces and sponge swab washings were enriched for O157 and the top 6 non-O157 STEC in EC broth (EMD Millipore Etobicoke, ON, Canada) at 37 °C for 6 h. Cultures were centrifuged (10 min at 6000× *g*) and DNA was extracted using a NucleoSpin Tissue kit (Macherey-Nagel, Düren, Germany) according to manufacturer’s instructions.

Targeted immunomagnetic separation (IMS; Romer Labs, Newark, DE, USA) with plating onto MacConkey (MAC) agar and sorbitol MAC agar containing Cefixime Tellurite (CT-SMAC) was conducted on PCR-positive cultures for retrieval of non-O157 and O157 cells, respectively [13]. Serogroup confirmation and the presence of s*tx1*, s*tx2*, *eae*, and *ehxA* virulence genes were determined by PCR [14]. Isolates were screened for motility (Hardy Diagnostics, Santa Maria, CA, USA), followed by flagellar (H-antigen) typing as previously described [15].

### 2.5. Generic E. coli Screening

Feces and pre-enriched swabs were streaked onto MAC agar and incubated overnight at 37 °C. Presumptive *E. coli* was streaked onto Tryptic Soy Agar (TSA) and incubated overnight at 37 °C, followed by testing for indole production (Ricca Chemical Co., Arlington, TX, USA). Lactose-fermenting and indole-producing isolates were screened via PCR for the presence of the *E. coli*-specific *uidA* gene [16,17].

### 2.6. Broad- and Extended-Spectrum β-Lactamase E. coli Screening

Feces and swab washings were added to EC broth containing cefotaxime (2 µg/mL) and incubated overnight at 37 °C. Enrichments were streaked onto MAC plates containing ceftriaxone (1 µg/mL) and incubated overnight at 37 °C. Typical *E. coli* colonies were streaked onto TSA containing ampicillin (32 µg/mL) and incubated overnight at 37 °C. Indole tests were completed as described above.

Broad- and extended-spectrum β-lactamase *E. coli* genotypes were determined by PCR for the presence of *bla*_CTX-M-1_-group, *bla*_CTX-M-2_-group, *bla*_CTX-M-9_-group, *bla*_OXA_, *bla*_SHV_, and *bla*_TEM_ as described previously [18]. PCR products were purified (Qiagen, Toronto, ON, Canada), sequenced (Eurofins Genomics, Louisville, KY, USA), and compared to ESBL-gene sequences within GenBank using BLAST.

### 2.7. Campylobacter Screening

In the second year, all fecal samples were screened for *Campylobacter* along with positive (*Campylobacter coli* ATCC 33559, *Campylobacter jejuni* ATCC 49943) and negative (*Escherichia coli* ATCC 25922) controls. Fecal slurries were added to Bolton broth containing selective supplement (BB+; Oxoid, Nepean, ON, Canada) and 5% laked horse blood (Oxoid, Nepean, ON, Canada). Enrichments were placed into an AnaeroJar with 2.5 L CampyGen sachets (Oxoid, Nepean, ON, Canada) to maintain microaerophilic conditions (5% O2, 10% CO_2_), and incubated for 2 h at 37 °C, followed by 20–22 h at 41.5 °C. As described previously [19], 100 µL of enriched BB+ culture was expelled onto a mixed cellulose ester membrane (0.65 μm; Millipore Ltd., Etobicoke, ON, Canada) placed on the surface of blood-free charcoal cefoperazone deoxycholate agar (CCDA+; Oxoid, Nepean, ON, Canada) containing selective supplement. After 15 ± 0.5 min, membranes were removed and discarded and CCDA+ plates were incubated for 24–48 h at 42 °C under microaerophilic conditions. Presumptive campylobacters were sub-cultured to blood agar (BD Canada, Mississauga, ON, Canada) containing 5% defibrinated sheep blood (Oxoid, Nepean, ON, Canada). To determine species (*C. coli* or *C. jejuni*), PCR assays were prepared using HotStar master mix (Qiagen, Toronto, ON, Canada) according to manufacturer’s instructions with a final primer concentration of 1000 nM [20]. *C. coli* and/or *C. jejuni* PCR-negative isolates were subjected to 16S rRNA PCR using primers 954F and 1369R [21,22] at a final concentration of 200 nM and sequenced (Eurofins Genomics, Toronto, ON, Canada).

### 2.8. Cryptosporidium and Giardia Screening

*Cryptosporidium* oocysts were extracted from fecal samples using the PowerFecal^®^ extraction kit (Qiagen, Toronto, ON, Canada). Briefly, 0.25 g of feces were suspended in buffers C1 and C2, followed by five freeze-thaw cycles, alternating between a dry ice/ethanol bath and 95 °C heating block. The remainder of the extraction followed manufacturer’s instructions. *Cryptosporidium* spp. were detected in the extracts by amplification of 18S rRNA as previously described [23] using Maxima Hot Start PCR Master Mix (Thermo Fisher Scientific, Toronto, ON, Canada) and final primer concentrations of 500 nM. Reactions were visualized on 1% agarose gels and 18S bands were extracted with the QIAquick gel extraction kit (Qiagen, Toronto, ON, Canada). Sanger sequencing was performed on 18S bands (Macrogen, Seoul, Korea). Genotype/species of *Cryptosporidium*-positive samples were identified based on the bioinformatic methods of Ruecker et al. [24].

A 292 bp fragment of the 16S small-subunit ribosomal RNA (SSU rRNA) was used to detect *Giardia intestinalis* and identification of genetic assemblages using endpoint PCR as described by Hopkins et al. [25] along with modifications as described by Smith et al. [26].

### 2.9. MRSA Screening

Alongside positive (*S. aureus* ATCC 43300) and negative (*S. aureus* ATCC 49775) controls, nasal swabs were transferred to SA broth (10 g tryptone/L, 75 g sodium chloride/L, 10 g mannitol/L and 2.5 g of yeast extract/L) and incubated at 35 °C for 24 h, then streaked onto MRSA CHROMagar plates (Dalynn Biologicals, Calgary, AB, Canada) and incubated at 35 °C for 24 h. Presumptive *S. aureus* colonies were transferred to 300 µL TSB and incubated at 35 °C for 24 h. Cultures were streaked onto MRSA CHROMagar prior to confirmation using the StaphTEX Blue latex agglutination test (Hardy Diagnostics, Santa Maria, CA, USA).

### 2.10. Antimicrobial Resistance Testing

A total of 583 *E. coli* isolates (425 generic, 99 putative-STEC, 59 ESBL-producing) and a control strain (*E. coli* ATCC 25922) were streaked onto TSA plates and incubated overnight at 37 °C. The direct colony method was used to prepare isolates for disk-diffusion testing according to CLSI M02-A12 [27]. The antibiotic panel (Sensi-disks, BD Canada) consisted of one or two representatives from eight classes (Figure 1). Disk-diffusion plate readings were completed using the BIOMIC V^3^ software version 7.1.1.2014 (Giles Scientific, Santa Barbara, CA, USA). Resistance was defined using established standards [28]. Multi-drug resistance (MDR) was defined as resistance ≥ 3 different classes of antibiotics [29].

### 2.11. E. coli Isolate Phylogroups

Phylogroup determination based on PCR results was completed for all *E. coli* isolates as described by Clermont et al. [30]. Briefly, an *E. coli* isolate was assigned to one of 8 phylogroups based on presence or absence of the genes *arpA*, *chuA*, *yjaA*, and *TspE4.C2*.

### 2.12. Statistical Analysis

Prevalence of STEC or *Campylobacter* spp. among sample types collected and animal species were compared with generalized linear mixed models (Proc Glimmix, SAS 9.3, SAS Institute Inc., Ottawa, ON, Canada) using a binomial distribution. Model adjusted means (back-transformed to original scale) were reported and deemed significant at *p* < 0.05.

## 3. Results

### 3.1. Survey

Survey responses are summarized in Table 1. The survey had a completion rate of 24% (187 completed questionnaires). Visitors were allowed food within the animal contact area at 51% of petting zoos and 71% of livestock pavilions at fairs. Reportedly, 31% and 23% of livestock pavilions and petting zoos, respectively, never sanitize any surfaces. Furthermore, nearly 30% of petting zoos and livestock pavilions did not provide hand-washing facilities or hand sanitizer. Only 2% of fairs reported an outbreak within the last 10 years; with the majority (75%) identifying *E. coli* as the causative agent.

### 3.2. Prevalence of O157 and Non-O157 STEC, Salmonella, Campylobacter, MRSA, Cryptosporidium and Giardia.

STEC O157 isolation frequency was lowest overall, isolated only from feces (0.6% of samples), while non-O157 STEC were isolated from all sources (22%), including hides/feathers and wooden hand rails (Table 2). Neither *Salmonella* nor MRSA were isolated from any samples. *Cryptosporidium* and *Giardia* were detected in 2% and 15% of fecal samples, respectively. *Campylobacter* spp. were the most prevalent and were isolated from 30% of fecal samples.

### 3.3. Screening for STEC and Related Genes

A viable putative-STEC isolate was obtained from 23% of samples. Following PCR detection of *stx1* and *stx2* toxin genes, 66 isolates were confirmed to be STEC. Serogroups O26 and O103 were observed at most petting zoos and were the most frequent overall (Table 3). Resistance to antibiotics, albeit to only one class, was most common for O26 isolates. Resistance was more likely for isolates of serogroup O26 than O103 (*p* < 0.05). The O26 isolates showing resistance to kanamycin and streptomycin were primarily isolated from petting zoo A, while O26 isolates resistant to tetracycline originated primarily from petting zoo F. Three MDR serotypes (O45:H21, O45:H19, O103:H2) from either feather or hand rail swabs, were also collected from petting zoos A and F. All O26 and O45 isolates contained *stx1,* while other virulence markers were variable across serogroups. All O145 isolates contained *eae* and *stx1* regardless of sample type or petting zoo. All O103:H38 isolates possessed *ehxA*, *eae*, and *stx1* genes regardless of host, sample type, phylogenetic group, or petting zoo.

### 3.4. Screening for Broad- and Extended-Spectrum β-Lactamase E. coli

Broad-spectrum β-lactamase (TEM-1) and ESBL (CTX-M) -producing *E. coli* (*n* = 59) were observed in 23.9% fecal samples. However, resistance determinants were only identified in 10.2% of these samples (21 isolates) (Table 4). TEM-1 was most common (*n* = 10), followed by CTX-M-15 (*n* = 8), and CTX-M-1 (*n* = 3).

### 3.5. Antimicrobial Resistance among Generic E. coli, Broad- and Spectrum β-Lastamase E. coli, and STEC

Antibiotic-resistant generic *E. coli* isolates were observed most often in pig (83%) and cattle (56%) feces, as well as feather swabs (66%; Table 5). The majority of AMR *E. coli* isolates from animal feces and hide/feather swabs (*n* = 69) were resistant to only one class of antibiotics. Six isolates were resistant to 3 or 4 classes. Isolates resistant ≥ 5 antibiotic classes were only obtained from cattle feces. Half of the AMR generic *E. coli* isolated from wooden hand rails were MDR. Conversely, AMR *E. coli* collected from metal hand rails were only resistant to tetracycline.

All broad- and extended-spectrum β-lastamase *E. coli* were isolated from animal feces, except for three MDR isolates from wooden hand rails (Table 5). More than 80% of the β-lastamase-producing *E. coli* were MDR, originating from pig (35%), sheep and goat (31%), and cattle (18%) feces.

Of the 66 STEC isolates collected in this study, 38% were resistant to antimicrobials (Table 5). All AMR STEC obtained from wooden hand rails were MDR, while those from feces and hides/feathers were resistant to only one class of antibiotic. One isolate from chicken feathers was the exception as it was resistant to five antibiotic classes.

### 3.6. Phylogroups among Generic E. coli and β-Lactamase-Producing E. coli

All *E. coli* phylogroups were represented among the generic and β-lactamase-producing *E. coli* isolates with the majority belonging to phylogroup B1 (Table 6). Phylogroup A was also consistently observed (11–13% of isolates) among all sample types. In addition to phylogroup B1, phylogroup E was also common in ESBL-producing *E. coli* (37%, Table 6). Isolates belonging to phylogroup C were observed among 0–6% of isolates from each sample type, while the remaining phylogroups (i.e., B2, D, F, and clades I/II) were uncommon (≤3%) among both generic and ESBL-producing *E. coli*.

## 4. Discussion

### 4.1. Survey

To our knowledge, this is the first survey of hygienic practices employed in Canadian livestock fairs and petting zoos. Several investigations from the U.S. have identified contact with animals and their feces, poor hand-washing compliance, and lack of or inadequately equipped hand-washing facilities as factors that precipitate disease outbreaks [31,32]. Restrictions on food and drink in animal areas, frequent and immediate removal of animal feces, provision of hand-washing and hand-sanitizing facilities, and more frequent surface disinfection are practices that could also reduce health risks in Canadian petting zoos and fairs. The U.S. National Association of State Public Health Veterinarians released a report of recommendations to prevent disease associated with animals in public settings [33]. A similar document outlining standard operating, cleaning, and sanitation procedures from a Canadian authority would provide a beneficial resource for agricultural fair coordinators and operators of public animal venues in Canada. However, without official policies and enforcement in place, compliance with recommendations remains voluntary.

### 4.2. Prevalence of O157 and Non-O157 STEC

Due to the low infective dose of STEC O157:H7 and the severity of illness that can develop, particularly in young children, it is the pathogen of utmost concern for petting zoos [2,5]. Previous studies reported a high prevalence of STEC O157 at county fairs in the U.S. (75–97%) with cattle being the primary host (11% host prevalence), followed by sheep and goats (3.6%), and swine (1.2%) [34]. In the current study, STEC O157 was isolated once from goat feces. Cattle were not common at the petting zoos sampled in this study; hence, only nine composite cattle fecal samples were collected compared to 36 from sheep and goats.

Pathogenic *E. coli* O157 can survive in the environment outside the animal host for weeks to months [4], and viable *E. coli* O157 have been cultured from fairgrounds and petting zoos months after outbreaks have occurred [35,36]. Hand rails can become contaminated at fairs and petting zoos and have been implicated as sources of *E. coli* O157 following outbreaks [36,37]. Although *E. coli* O157 was not isolated from hand rail swabs in the current study, non-O157 STEC were, even from hand rails that were reportedly sanitized daily.

To our knowledge, this is the first study to examine the prevalence of the top 6 non-O157 STEC serogroups in animals at fairs and petting zoos. Most studies of non-O157 STEC report prevalence in food animals, of which cattle, small ruminants, pigs, and poultry are important sources [5,38]. However, non-O157 STEC are widespread and have been isolated from numerous non-food domestic and wild animals [5,32]. According to previous studies, llamas, horses, and donkeys are not considered important STEC reservoirs [4,39]. In the current study, STEC were isolated from llama feces from the same petting zoo on two separate occasions, and from horse and donkey feces collected from two different petting zoos (Table 2). The relatively high prevalence of STEC in fecal samples from these animals may reflect animal-to-animal transmission, considering the proximity of housing within petting zoos and fairs [40]. This possibility is supported by the isolation of STEC from multiple animal species at the same petting zoos that possessed the same serotype, virulence, and AMR phenotypes.

Research of STEC loads on hides is primarily focused on cattle since hides represent a major source of carcass contamination during meat processing [5]. No research comparable to the current study for STEC prevalence on the hides, fur, or feathers of other animal species could be identified. Considering that contact with animals at petting zoos is primarily from active contact with hides and feather, findings of the current study emphasize the importance of supplying hand-washing facilities within petting zoo areas.

### 4.3. Prevalence of Salmonella, MRSA, Campylobacter, Cryptosporidium and Giardia

Research suggests that the prevalence of *Salmonella* is lower in Canada and the northern U.S. than the southern U.S. [41,42] and that management practices may have a profound effect on the transmission and persistence of salmonellae within an integrated production system [43]. Animals sampled within this study were not a part of the production system, coupled with the small sample size, the lack of *Salmonella* in this study was perhaps not surprising.

Pigs are a reservoir for *S. aureus*, and MRSA have been isolated from pigs at an agricultural fair in the U.S. [44]. Though pigs at fairs in Canada have not previously been tested, other studies indicate MRSA are common in pigs on Canadian production farms [45]. Since pigs were not frequently encountered at the petting zoos visited in this study resulting in a small sample size, it is perhaps not surprising that none of the nasal swabs were positive for MRSA.

Compared to the other pathogens in this study, *Campylobacter* is thought to cause the highest number of human illnesses due to transmission from animals [3]. Healthy poultry, cattle, swine, and sheep are considered important reservoirs for this organism [20,46]. We isolated *Campylobacter* spp. from sheep and goats, pigs, horses and donkeys, and birds. Isolation of *Campylobacter* from healthy horses has been reported, but much less frequently than for other host species [47]. A similar study isolated *Campylobacter* spp. from cattle, sheep, goats, and chickens at a petting zoo in California [11]. In contrast to the high proportion of *Campylobacter*-positive samples found in our study, a meta-analysis estimate of *Campylobacter* prevalence among petting zoo animals was determined to be 6.5% [48]. The meta-analysis was derived from only seven studies, as there is a lack of research on *Campylobacter* at fairs and petting zoos in Canada. Furthermore, non-*jejuni*/*coli* species are of uncertain significance to human health [46]. Additional work is required to fully assess the risk of *Campylobacter* infection from petting zoos and fairs in Canada.

*Cryptosporidium* was only detected in sheep and goat feces, with the *Cryptosporidium bovis* identified in goat/sheep fecal samples occasionally being associated with human illness [49]. A zoonotic assemblage of *Giardia intestinalis* (assemblage A) was detected in cattle, horses, and donkeys. Neither pathogen was detected in feces of petting zoo animals in a similar U.S. study [11].

### 4.4. AMR, Broad- and Extended-Spectrum β-Lactamase- E. coli

AMR *E. coli* were observed among all animal groups and sample types tested. Collectively, 22% of the generic *E. coli* isolates from this study were resistant to one or more antimicrobials. Other studies report varied rates of resistance in generic *E. coli* from different animal groups in Canada [50,51].

Very few isolates (1.3%) were resistant to the critically important 3rd generation cephalosporins or quinolones. No isolates were resistant to imipenem. Resistance to tetracycline was most common and has been widely reported in dairy and beef cattle, small ruminants, pigs, poultry, equines, and petting zoo animals [11,51,52,53,54]. Resistance to ampicillin, streptomycin and kanamycin is also common in *E. coli* from animal feces [11,52,54].

Although MDR among generic *E. coli* and STEC was not common, MDR *E. coli* including STEC were isolated from wooden hand rails, possibly posing an even greater risk of transmission within the petting zoo than bacteria directly associated with animal feces. Although these isolates may not be pathogenic, they may be carriers of AMR genes that can be readily exchanged with other bacteria [50].

Since the late 1990s, ESBL-producing *E. coli* have emerged globally [10]. Early β-lactamase-producing *E. coli* possessed mainly TEM and SHV β-lactamases, while the significance of CTX-M-type enzymes has since increased and currently represents the most common type in humans [10]. Infection with β-lactamase-producing *E. coli* is of concern because of limited therapeutic options [10]. Only the most common broad-spectrum β-lactamase and ESBL genotypes were screened for in this study, suggesting the isolates with unidentified genotypes (>60%) may have been of less common β-lactamase types. The detection of broad- and extended-spectrum β-lactamase-producing *E. coli* in this study is to be considered a preliminary screening. Future studies must also include phenotypic confirmation of putative ESBL-producing *E. coli* isolates.

### 4.5. Phylogroups

The clonal population structure of *E. coli* has enabled assignation of isolates to phylogroups (A, B1, B2, C, D, E, F, and cryptic clades) [29,30] which vary in host association, presence of virulence factors, and persistence in the non-host environment [29]. Distinct ecological niches characterize the different phylogroups. For example, phylogroups A and B2 are predominant in humans, whereas phylogroups A and B1 are more common in animals [55].

Although representatives from every phylogroup were identified, the most common phylogroup was B1. These results are consistent with previous studies which have identified a higher proportion of B1 among cows, goats, and sheep [56]. Our results suggest that *E. coli* from phylogroups A, B1, and E are the most common in petting zoo animals and their environments. From a human health perspective, *E. coli* responsible for mild and chronic diarrhea are found throughout the phylogenetic tree, whereas *E. coli* that cause more severe pathologies are frequently restricted to phylogroups A, B1, and E [55]. Whether any of the isolates collected in the current study could cause mild or severe illness in humans remains unknown, but measures that reduce the risk of ingestion of these bacteria [2], as well as cleaning and sanitizing procedures to decrease environmental persistence undoubtedly have merit.

## 5. Conclusions

Effective hygienic preventative measures within fairs and petting zoos rely on assessing risks for human exposure to zoonotic pathogens. Despite the limited number of petting zoos and samples in this study, we have shown that some zoonotic pathogens are clearly present within petting zoo animals and their environments. Of concern are STEC and Campylobacter species based on their prevalence and/or potential to cause serious illness. In addition, a variety of virulence genes and AMR *E. coli* were present in a variety of animal species and on hand rails suggesting the potential for transfer of these elements to other *E. coli* and/or pathogens.

Certain inadequacies are evident within the operation of animal areas accessible to the public at fairs in Canada, such as the absence of hand-washing facilities at many venues. Sanitization of surfaces within petting zoos and livestock pavilions is largely sporadic, with some venues lacking sanitization procedures. These data should not deter the public from visiting agricultural fairs but should serve as reminder to event operators and visitors alike, of the importance of good hygiene and sanitation practices. Materials used to construct animal areas and pens should be non-porous, such as metal rather than wood, allowing more effective sanitization. Fair and petting zoo operators are encouraged to consult the recommendations outlined by the National Association of State Public Health Veterinarians. Ongoing efforts to reduce risks of human exposure to zoonotic pathogens can help to ensure children and adults continue to enjoy agricultural events and the educational opportunities they provide.

Further research is necessary to better understand the human health risk associated with fairs and petting zoos. Future studies should include identification of plasmids in *E. coli* strains to identify the dissemination potential and possible transfer of AMR genes to other *E. coli* strains and other members of Enterobacteriaceae. Multilocus Sequence Typing (MLST) of *E. coli* strains could be used to determine clonality and animal/human dissemination potential. Additionally, the inclusion of animal and human clinical isolates could be used to identify common characteristics between clinical isolates and those collected from petting zoos.

## Figures and Tables

**Table 1:** Summary of the survey results concerning livestock pavilions and petting zoos at fairs across Canada.

Question	Answer	% Fairs	
How many visitors attend the fair each year? (*n* = 185)	under 5000 visitors	36	
	5000–19,999 visitors	36	
	20,000–99,999 visitors	20	
	100,000 and over	8	
Where is the fair located? (*n* = 185)	Alberta	14	
	British Columbia	11	
	Manitoba	5	
	Ontario	49	
	Quebec	9	
	Other	12	
Is there a petting zoo featured at the fair? (*n* = 171)	Yes	71	
Is there a livestock pavilion or barn? (*n* = 184)	Yes	72	
Has there been an outbreak or problem with a pathogen in the past 10 years? (*n* = 164)	Yes	2	
What was the pathogen of concern? (among those that reported a problem or outbreak) (*n* = 4)	*E. coli*	75	
	Unknown	25	
**Question**	**Answer**	**% Livestock Pavilions at fairs**	**% Petting Zoos at fairs**
Which animal species are present at the facility? (L *n* = 131; P *n* = 119)	Cattle and Calves	89	44
	Horses and Ponies	77	71
	Sheep	69	72
	Goats	55	78
	Rabbits	62	73
	Pigs	40	52
	Donkeys	NA	52
	Alpacas or Llamas	NA	36
	Guinea Pigs	NA	19
What is the level of contact between humans and animals within petting zoos? (*n* = 117)	Animals roam freely without barriers and visitors can pet, hold, and feed the animals	NA	17
	Animals roam freely without barriers visitors can pet and hold the animals	NA	5
	A barrier separates animals and visitors, and visitors can pet and feed the animals	NA	44
	A barrier separates animals and visitors, and visitors can only pet the animals	NA	34
What is the level of contact between humans and animals at the livestock pavilion? (*n* = 131)	None, only exhibitors have contact with the animals	11	NA
	Public is permitted to walk through the livestock area	71	NA
	Public is encouraged to touch or interact with the animals	18	NA
Is there a children’s play area directly adjacent? (L *n* = 130; P *n* = 115)	Yes	18	30
Is there a food or beverage area directly adjacent? (L *n* = 131; P *n* = 116)	Yes	16	2
Are there rules regarding visitors bringing food or drink into the animal area? (L *n* = 125; P *n* = 117)	Not allowed	29	49
	Allowed, but no touching animals	7	5
	Allowed, not for feeding animals	25	25
	No restrictions	39	21
Is there continuous removal of feces? (L *n* = 103; P *n* = 84)	Yes	78	69
What is the frequency of removal of feces from the animal area? (L *n* = 117; P *n* = 111)	Immediately	57	60
	2–4 times daily	32	32
	Once daily	8	8
	Less frequently	3	0
Are there hand-washing stations? (L *n* = 123; P *n* = 117)	Yes	73	69
Are there hand sanitizers? (L *n* = 96; P *n* = 96)	Yes	73	79
Does the staff remind visitors to wash their hands? (L *n* = 117; P *n* = 111)	Yes	44	69
Is there hand-washing signage? (L *n* = 97; P *n* = 88)	Yes	73	72
Is there no smoking signage? (L *n* = 89; P *n* = 63)	Yes	67	52
Is there no eating signage? (L *n* = 39; P *n* = 43)	Yes	30	35
How often are surfaces sanitized? (L *n* = 111; P *n* = 107)	Multiple times a day	18	30
	Once daily	19	20
	Only when visibly dirty	32	27
	Never	31	23
Is there a safe area for storage of visitor items? (L *n* = 117; P *n* = 113)	Yes	25	30

NA = not applicable. L = Livestock Pavilions. P = Petting Zoos.

**Table 2:** Prevalence of O157 and the top 6 non-O157 STEC, *Salmonella*, *Campylobacter* spp., *Cryptosporidium* and *Giardia* from samples collected at petting zoos in Canada (2015–2016).

Sample Type	Host	% STEC		% *Salmonella*	% *Campylobacter* ^1^			% *Cryptosporidium*	% *Giardia* ^6^
		O157	Non-O157		*jejuni*	*coli*	*lanienae*		
Composite Fecal Samples	Cattle	0 (0/9)	44 (4/9)	0 (0/9)	0 (0/5)	0 (0/5)	0 (0/5)	0 (0/9)	22 (2/9)
	Sheep and Goats	3 (1/36)	19 (7/36)	0 (0/36)	7 (1/15)	7 (1/15)	0 (0/15)	6 (2/36) ^5^	25 (9/36)
	Pigs	0 (0/12)	33 (4/12)	0 (0/12)	0 (0/10)	20 (2/10)	30 (3/10)	0 (0/12)	0 (0/12)
	Horses and Donkeys	0 (0/18)	28 (5/18)	0 (0/18)	0 (0/8)	38 (3/8) ^2^	25 (2/8) ^2^	0 (0/18)	11 (2/18)
	Llamas	0 (0/6)	100 (6/6)	0 (0/6)	NT ^4^	NT ^4^	NT ^4^	0 (0/6)	0 (0/6)
	Birds ^3^	0 (0/7)	29 (2/7)	0 (0/7)	17 (1/6)	17 (1/6)	0 (0/6)	0 (0/7)	0 (0/7)
**Total**		**1 (1/88)**	**32 (28/88)**	**0 (0/88)**	**5 (2/44)**	**16 (7/44)**	**11 (5/44)**	**2 (2/88)**	**15 (13/88)**
Hide/Feather Swabs	Cattle	0 (0/4)	0 (0/4)	0 (0/4)	NT ^4^	NT ^4^	NT ^4^	NT ^4^	NT ^4^
	Sheep and Goats	0 (0/15)	7 (1/15)	0 (0/15)	NT ^4^	NT ^4^	NT ^4^	NT ^4^	NT ^4^
	Pigs	0 (0/5)	20 (1/5)	0 (0/5)	NT ^4^	NT ^4^	NT ^4^	NT ^4^	NT ^4^
	Horses and Donkeys	0 (0/5)	0 (0/5)	0 (0/5)	NT ^4^	NT ^4^	NT ^4^	NT ^4^	NT ^4^
	Llamas	0 (0/3)	0 (0/3)	0 (0/3)	NT ^4^	NT ^4^	NT ^4^	NT ^4^	NT ^4^
	Birds ^3^	0 (0/3)	100 (3/3)	0 (0/3)	NT ^4^	NT ^4^	NT ^4^	NT ^4^	NT ^4^
	Rabbits	0 (0/1)	0 (0/1)	0 (0/1)	NT ^4^	NT ^4^	NT ^4^	NT ^4^	NT ^4^
**Total**		**0 (0/36)**	**14 (5/36)**	**0 (0/36)**					
Hand Rail Swabs	Wood	0 (0/28)	14 (4/28)	0 (0/28)	NT ^4^	NT ^4^	NT ^4^	NT ^4^	NT ^4^
	Metal	0 (0/18)	0 (0/18)	0 (0/18)	NT ^4^	NT ^4^	NT ^4^	NT ^4^	NT ^4^
**Total**		**0 (0/46)**	**9 (4/46)**	**0 (0/46)**					
**Grand Total**		**0.6 (1/170)**	**22 (37/170)**	**0 (0/170)**	**5 (2/44)**	**16 (7/44)**	**11 (5/44)**	**2 (2/88) ^5^**	**15 (13/88) ^6^**

Prevalence % (# positive samples/# samples collected). ^1^ Campylobacter was only isolated from feces in the second year. ^2^ 1 C. coli and 1 C. lanienae collected from the same sample. ^3^ Birds = Chickens, ducks, peacocks, turkeys, parakeets. ^4^ NT = Not tested. ^5^ 1 C. bovis, and 1 C. xiaoi. ^6^ All G. intestinalis assemblage A.

**Table 3:** Characteristics of STEC isolates (*n* = 66).

Serogroup	H-Type	Motility ^1^	Petting Zoo ID ^2^	Sample Type	Host	Virulence Markers	Phylogenetic Group ^3^	# of Isolates	Antibiotic Resistance Pattern (# Antibiotic Classes) ^4,5^
O26	H34	+	A2	feces	cattle	ehxA, eae, stx1	U	1	NA
O26	H46	+	A2	feces	cattle	stx1	U	1	KAN-STREP (1)
O26	H46	+	A2	feces	cattle	ehxA, stx1	U	1	KAN-STREP (1)
O26	H34	+	A2	feces	cattle	stx1	Multiple ^6^	2	NA
O26	H46	+	F	feces	donkey	ehxA, eae, stx1, stx2	A	1	TET (1)
O26	H46	+	A1	feces	donkey	eae, stx1	U	1	KAN-STREP (1)
O26	H46	+	A1	feces	donkey	ehxA, stx1	U	1	KAN-STREP (1)
O26	H46	+	A1, F	feces	donkey	stx1	U	4^7^	KAN-STREP (1)
O26	H46	+	A2	feces	donkey	stx1	A	2	TET (1)
O26	H11	+	C1	feces	goat	ehxA, eae, stx1	U	2^7^	NA
O26	H46	+	A2	feces	goat	eae, stx1	U	1	KAN-STREP (1)
O26	H46	+	F	feces	goat	stx1, stx2	A	1	TET (1)
O26	H46	+	F	feces	horse	eae, stx1, stx2	A	1	TET (1)
O26	H34	+	A2	feces	llama	stx1	B2	2^7^	NA
O26	H46	+	F	feces	pig	stx1, stx2	A	3^7^	TET (1)
O26	H46	+	F	feather swab	chicken	stx1	A	1	TET (1)
O26	H46	+	F	hand rail	wood	stx1	A	1	NA
O45	H34	+	A2	feces	llama	eae, stx1	U	1	NA
O45	H21	NM	F	hand rail	wood	ehxA, eae, stx1	U	1	CHL-STREP-TET-TMS (4)
O45	H19	NM	F	feather swab	chicken	stx1	E	1	AMC-AMP-CAZ-CFX-CHL-CRO-CTX-GEN-STREP-TET (5)
O103	H43	+	B	feces	cattle	ehxA, eae, stx2	B1	1	NA
O103	H43	+	B	feces	cattle	stx2	U	1	NA
O103	H43	+	B	feces	cattle	stx1	B1	1	NA
O103	H38	+	F	feces	donkey	ehxA, eae, stx1	B1	2	NA
O103	H38	+	F	feces	duck	ehxA, eae, stx1	Multiple ^8^	3 ^7^	NA
O103	H38	+	F	feces	goat	ehxA, eae, stx1	B1	2	NA
O103	H43	+	B	feces	sheep or goat	stx2	B1	1	NA
O103	H43	+	B	feces	sheep or goat	stx1	E	1	NA
O103	H19	+	B	feces	sheep or goat	stx2	U	1	NA
O103	H38	+	F	feces	horse	ehxA, eae, stx1	B1	1	NA
O103	H21	+	F	feces	horse	stx1	B1	1	NA
O103	H38	+	F	feces	pig	ehxA, eae, stx1	B1	1	NA
O103	H19	+	C2	feces	pig	stx2	B1	1	NA
O103	H2	+	A2	hand rail	wood	eae, stx1	Multiple ^9^	3	STREP-TET-TMS (3)
O103	H4	+	F, C2	hand rail	wood	eae, stx1	B1	3 ^7^	NA
O103	H42	+	C2	hand rail	wood	eae, stx1	B1	1	NA
O103	H38	+	F	feather swab	duck	ehxA, eae, stx1	B1	1	NA
O103	H38	+	C1	hide swab	goat	ehxA, eae, stx1	E	1	TET (1)
O121	H19	+	A1	feces	llama	eae, stx2	B1	1	NA
O121	H7	+	C2	feces	pig	stx1	B1	1	TET (1)
O145	H25	+	A1	feces	llama	eae, stx1	Multiple ^10^	3 ^7^	NA
O145	H28	NM	F	feces	pig	eae, stx1	E	1	NA
O145	H25	+	E	hide swab	pig	eae, stx1	B2	2	NA
O157	H7	+	C1	feces	goat	ehxA, eae, stx1, stx2	E	3	NA

^1^ Motility test (+ = motile; NM = non-motile). ^2^ Petting zoos sampled: A1 (1^st^ sampling), A2 (2^nd^ sampling), B, C1 (1^st^ sampling), C2 (2^nd^ sampling), D, E, and F. ^3^ U = unknown. ^4^ NA = Not applicable. ^5^ CHL, chloramphenicol; KAN, kanamycin; STREP, streptomycin; TET, tetracycline; TMS, trimethoprim/sulfamethoxazole. ^6^ 1 isolate = clade I; 1 isolate = B2. ^7^ Isolates collected from different samples. ^8^ 1 isolate B1, 2 isolates = U. ^9^ 1 isolate = U; 1 isolate = B1; 1 isolate = E. 1 isolate = U; 2 isolates = B2.

**Table 4:** Distribution of phylogenetic subgroups among broad- and extended-spectrum β-lactamase *E. coli* genotypes isolated from the feces of petting zoo animals (*n* = 9).

ESBL Genotype	Animal Group	Phylogenetic Group ^1^	# Isolates
bla_CTX-M-1_	cattle	U	3
bla_CTX-M-15_	birds	A	1
	birds	B1	1
	birds	U	1
	goats	B1	3
	pigs	B1	2
bla_TEM-1_	cattle	B1	4
	cattle	E	1
	cattle	U	1
	donkeys	F	1
	pigs	E	3

^1^ U = unknown.

**Table 5:** Frequency of antimicrobial resistance (AMR) patterns among generic *E. coli* (*n* = 425), broad- and extended-spectrum β-lactamase *E. coli* (*n* = 59), and STEC (*n* = 66) isolates from animal feces, hide/feather swabs, and hand rails.

	Composite Fecal Samples						Hide or Feather Swabs							Hand Rail Swabs
AMR Pattern (# Antibiotic Classes) ^1^	Cattle	Sheep/Goats	Pigs	Horses/Donkeys	Llamas	Birds	Cattle	Sheep/Goats	Pigs	Horses/Donkeys	Llamas	Birds	Wood	Metal
*Generic E. coli (n= # isolates)*	*n* = 25	*n* = 108	*n* = 35	*n* = 52	*n* = 18	*n* = 18	*n* = 12	*n* = 33	*n* = 8	*n* = 14	*n* = 9	*n* = 7	*n* = 66	*n* = 20
AMC-AMP-CAZ-CFX-CHL-CRO-CTX-GEN-KAN-STREP-TET-TMS (6)	1 ^2^	-	-	-	-	-	-	-	-	-	-	-	-	-
AMC-AMP-CAZ-CFX-CHL-CRO-CTX-KAN-STREP-TET-TMS (6)	2	-	-	-	-	-	-	-	-	-	-	-	-	-
AMC-AMP-CAZ-CFX-CHL-CRO-CTX-GEN-KAN-STREP-TET (5)	2	-	-	-	-	-	-	-	-	-	-	-	-	-
AMC-AMP-KAN-STREP-TET (4)	-	3	-	-	-	-	-	-	-	-	-	-	-	-
AMP-STREP-TET-TMS (4)	-	-	-	-	1	-	-	-	-	-	-	-	4	-
CHL-STREP-TET-TMS (4)	-	-	-	-	-	-	-	-	-	-	-	-	1	-
CHL-STREP-TET (3)	-	-	1 ^3^	-	-	-	-	-	-	-	-	-	2	-
CHL-STREP-TMS (3)	-	-	-	1 ^4^	-	-	-	-	-	-	-	-	-	-
CTX-CRO (1)	-	-	-	-	-	-	-	-	-	-	1	-	-	-
KAN-STREP (1)	-	1	-	3	-	-	-	-	-	-	-	-	-	-
STREP-TET (2)	1	3	6	-	-	-	-	1	-	2	-	2	-	-
STREP-TMS (2)	-	-	-	1	-	-	-	-	-	-	-	-	-	-
TET-TMS (2)	-	-	1	-	-	-	-	-	-	-	-	-	-	-
STREP (1)	-	1	5	-	-	-	-	-	-	-	-	-	-	-
TET (1)	2	10	2	4	-	5	-	9	1	1	-	-	7	3
AMP (1)	1	-	1	-	4	-	-	1	-	-	-	-	-	-
Susceptible isolates	16	90	19	43	13	13	0	22	7	11	8	5	52	17
General resistance prevalence per sample (%) ^5^	**5/9 (56)**	**9/36 (25)**	**10/12 (83)**	**4/18 (22)**	**3/6 (50)**	**3/7 (43)**	**0/4 (0)**	**5/15 (33)**	**1/5 (20)**	**2/5 (40)**	**1/3 (33)**	**2/3 (66)**	**8/28 (29)**	**1/18 (6)**
AMC-AMP-CAZ-CFX-CHL-CIP-CRO-CTX-NAL-STREP-TET-TMS (7)	-	1	-	-	-	-	-	-	-	-	-	-	-	-
AMC-AMP-CAZ-CFX-CHL-CRO-CTX-GEN-KAN-STREP-TET (5)	6	-	-	-	-	-	-	-	-	-	-	-	-	-
AMP-CAZ-CFX-CHL-CIP-CRO-CTX-NAL-STREP-TET-TMS (6)	-	1	-	-	-	-	-	-	-	-	-	-	-	-
AMC-AMP-CAZ-CFX-CHL-CRO-CTX-NAL-STREP-TET (6)	-	6	-	-	-	-	-	-	-	-	-	-	3	-
AMC-AMP-CAZ-CFX-CHL-CRO-CTX-STREP-TET-TMS (6)	-	3	4	-	-	-	-	-	-	-	-	-	-	-
AMP-CAZ-CHL-CIP-CRO-CTX-NAL-STREP-TET-TMS (6)	-	-	1 ^6^	-	-	-	-	-	-	-	-	-	-	-
AMC-AMP-CAZ-CFX-CHL-CRO-CTX-STREP-TET (5)	3	3	2	3	-	-	-	-	-	-	-	-	-	-
AMP-CHL-CIP-CRO-CTX-NAL-STREP-TET-TMS (6)	-	1	2	-	-	2	-	-	-	-	-	-	-	-
AMC-AMP-CAZ-CFX-CRO-CTX-STREP-TET (4)	-	-	2	-	-	-	-	-	-	-	-	-	-	-
AMP-CAZ-CHL-CRO-CTX-STREP-TET-TMS (5)	-	-	1	-	-	-	-	-	-	-	-	-	-	-
AMC-AMP-CAZ-CFX-CRO-CTX-TET (3)	-	-	4	-	-	-	-	-	-	-	-	-	-	-
AMC-AMP-CFX-CHL-CTX-STREP-TET (5)	-	-	1	-	-	-	-	-	-	-	-	-	-	-
AMC-AMP-CAZ-CFX-CRO-CTX (2)	-	6	-	-	-	-	-	-	-	-	-	-	-	-
AMP-CRO-CTX-TET (2)	-	-	-	-	-	1	-	-	-	-	-	-	-	-
AMP-CRO-CTX (1)	3	-	-	-	-	-	-	-	-	-	-	-	-	-
General resistance prevalence per sample (%) ^5^	**4/9 (44)**	**7/36 (19)**	**7/12 (58)**	**1/18 (6)**	**0/0 (0)**	**1/7 (14)**	**0/0 (0)**	**0/0 (0)**	**0/0 (0)**	**0/0 (0)**	**0/0 (0)**	**0/0 (0)**	**1/28 (4)**	**0/0 (0)**
AMC-AMP-CAZ-CFX-CHL-CRO-CTX-GEN-STREP-TET (5)	-	-	-	-	-	-	-	-	-	-	-	1	-	-
CHL-STREP-TET-TMS (4)	-	-	-	-	-	-	-	-	-	-	-	-	1	-
STREP-TET-TMS (3)	-	-	-	-	-	-	-	-	-	-	-	-	3	-
KAN-STREP (1)	2	1	-	6	-	-	-	-	-	-	-	-	-	-
TET (1)	-	1	4	4	-	-	-	1	-	-	-	1	-	-
Susceptible isolates	6	10	3	4	7	3	0	0	2	0	0	1	5	0
General resistance prevalence per sample (%) ^5^	**1/9 (11)**	**2/36 (6)**	**3/12 (25)**	**5/18 (28)**	**0/6 (0)**	**0/7 (0)**	**0/4 (0)**	**1/15 (7)**	**0/5 (0)**	**0/5 (0)**	**0/3 (0)**	**2/3 (66)**	**2/28 (7)**	**0/18 (0)**

^1^ AMC, amoxicillin/clavulonic acid; AMP, ampicillin; CAZ, ceftazidime; CHL, chloramphenicol; CIP, ciprofloxacin; CFX, cefoxitin; CRO, ceftriaxone; CTX, cefotaxime; GEN, gentamycin; KAN, kanamycin; NAL, naladixic acid; STREP, streptomycin; TET, tetracycline; TMS, trimethoprim/sulfamethoxazole. ^2^ Intermediate resistance to CAZ and GEN. ^3^ Intermediate resistance to STREP. ^4^ Intermediate resistance to CHL and TMS. ^5^ # samples with ≥1 AMR isolate /total # samples. ^6^ Intermediate resistance to CAZ.

**Table 6:** Assigned phylogenetic groups (A, B1, B2, C, D, E, F, Clades I or II, or Unknown) of *E. coli* strains from different hosts based on multiplex PCR of 4 genetic markers (*arpA*, *chuA*, *yjaA*, TspE4.C2). Numbers indicate the number of bacterial isolates obtained per species. An overall representation of bacterial isolates per phylogroup is given as a percentage out of the total number of isolates collected.

	Phylogroup	Cattle	Sheep/Goats	Pigs	Horses/Donkeys	Llamas	Birds	Wood	Metal	% Isolates
Generic *E. coli* isolates from animal feces (*n* = 256)	A	5	15	5	1	1	2	NA	NA	11
	B1	17	67	25	33	13	13	NA	NA	66
	B2	0	0	0	0	0	0	NA	NA	0
	C	0	7	0	7	0	1	NA	NA	6
	D	0	1	1	3	0	0	NA	NA	2
	E	2	7	2	3	4	1	NA	NA	7
	F	0	0	0	0	0	0	NA	NA	0
	Clade I or II	0	3	1	0	0	0	NA	NA	2
	Unknown	1	8	1	5	0	1	NA	NA	6
Generic *E. coli* isolates from animal hide swabs (*n* = 83)	A	1	1	1	0	0	7	NA	NA	12
	B1	8	23	6	12	5	0	NA	NA	65
	B2	0	0	0	0	2	0	NA	NA	2
	C	1	3	1	0	0	0	NA	NA	6
	D	0	0	0	1	0	0	NA	NA	1
	E	1	3	0	1	2	0	NA	NA	8
	F	0	0	0	0	0	0	NA	NA	0
	Clade I or II	0	1	0	0	0	0	NA	NA	1
	Unknown	1	2	0	0	0	0	NA	NA	4
Generic *E. coli* isolates from hand rail swabs (*n* = 86)	A	NA	NA	NA	NA	NA	NA	8	3	13
	B1	NA	NA	NA	NA	NA	NA	39	12	59
	B2	NA	NA	NA	NA	NA	NA	0	0	0
	C	NA	NA	NA	NA	NA	NA	2	0	2
	D	NA	NA	NA	NA	NA	NA	0	0	0
	E	NA	NA	NA	NA	NA	NA	14	5	22
	F	NA	NA	NA	NA	NA	NA	0	0	0
	Clade I or II	NA	NA	NA	NA	NA	NA	3	0	3
	Unknown	NA	NA	NA	NA	NA	NA	0	0	0
B-lactamase -producing *E. coli* isolates from feces and hand rail swabs (*n* = 59)	A	0	0	3	0	0	1	0	0	7
	B1	4	12	7	0	0	1	0	0	41
	B2	0	0	0	0	0	0	0	0	0
	C	0	0	0	0	0	0	0	0	0
	D	0	0	1	0	0	0	0	0	1.5
	E	4	9	3	3	0	0	3	0	37
	F	0	0	0	1	0	0	0	0	1.5
	Clade I or II	0	0	0	0	0	0	0	0	0
	Unknown	4	0	2	0	0	1	0	0	12
